# Higher Perceived Stress and Poor Glycemic Changes in Prediabetics and Diabetics Among Indian Population

**DOI:** 10.25122/jml-2019-0055

**Published:** 2020

**Authors:** Amit Mishra, Vivek Podder, Shweta Modgil, Radhika Khosla, Akshay Anand, Raghuram Nagarathna, Rama Malhotra, Hongasandra Ramarao Nagendra

**Affiliations:** 1. Department of Yoga and Life Sciences, Swami Vivekananda Yoga Anusandhana Samsthana, Bangalore, India; 2. Department of General Medicine, Kamineni Institute of Medical Sciences, Narketpally, Nalgonda, India; 3. Neuroscience Research Lab, Department of Neurology, Postgraduate Institute of Medical Education and Research, Chandigarh, India; 4. Department of Psychiatry, Postgraduate Institute of Medical Education and Research, Chandigarh, India

**Keywords:** Diabetes mellitus, perceived stress, HbA1c, prediabetes, CCRYN (Central Council of Research in Yoga and Naturopathy, New Delhi), DM (Diabetes mellitus), FBS (fasting blood sugar), HbA1c (glycated hemoglobin), HPA (hypothalamic-pituitary-adrenal axis), IDRS (Indian Diabetes Risk score), IYN (Indian Yoga Association), NMB (Niyantrita Madhumeha Bharata Abhiyaan), PPBS (postprandial blood sugar), PSS (Perceived Stress Scale), WHO (World Health Organization)

## Abstract

Diabetes mellitus (DM) is a chronic metabolic disorder with significant co-morbidities and healthcare burdens. Many large studies have investigated the association between perceived stress and DM; however, none investigated this in a larger Indian population.

We hypothesized stress as one of the reasons for the progression of people with prediabetes into DM. The present study was, therefore, planned to report on associations between perceived stress and blood glucose markers stratified by diabetic status.

The current descriptive study was a subset analysis of the nationwide cross-sectional survey, conducted in all Indian zones under the National Multicentric Diabetes Control Program. The study examined the perceived stress levels using a perceived stress scale (PSS-10) in people with prediabetes (n=649) and DM (n=485) and then segregated them into three categories (minimum, moderate, and severe). Blood glucose markers (fasting blood glucose, postprandial blood glucose, and HbA1c) were evaluated to report their association with the perceived stress. The study revealed a significantly higher HbA1c level in people with prediabetes, particularly those with severe perceived stress (6.12 ± 0.27) compared to other categories. Those with DM had a higher fasting blood glucose level, particularly with severe perceived stress (239.28 ± 99.52). An increased HbA1c level is noted in severely stressed people with prediabetes, requiring a comprehensive analysis with a longitudinal study of the role of perceived stress in the progression of prediabetes into DM. Additionally, higher fasting blood glucose levels in patients with DM and severe perceived stress suggests the need for establishing comprehensive diabetic care inclusive of stress management.

## Introduction

Diabetes mellitus (DM) is a chronic metabolic disorder with significant co-morbidities and healthcare burdens. In a previous study conducted by SendhilKumar et al., the prevalence of DM in India was reported to be 7.3% [1]. As per the current national survey, approximately 70 million Indians suffer from DM with an anticipated rise to 120.5 million by 2040 [2].

According to the World Health Organization (WHO) report, the global prevalence of DM was 4.0% in 1993, with developed countries being affected more. Among various countries, the Indian and Chinese populations score higher [3]. This prevalence rate has drastically increased to 8.8% in 2015 [4]. A multinational, large global estimation study for DM in 2016 found that there is a four-fold increase in the number of people with DM between 1980 and 2014, with the age-standardized prevalence among men and women increased by more than 50% [5]. The significant risk factors associated with DM include non-modifiable (family history and age) and modifiable (obesity, sedentary lifestyle, lack of physical exercise, and unbalanced diet) factors. India, being an overpopulated and developing country, has more people in the age group of 60–79 years, thus increasing the DM risk in the population [6]. Despite worldwide efforts to increase treatment, screening, and preventive programs, DM prevalence continues to increase.

Factors like stress, anxiety, and depression play a crucial role in influencing this metabolic problem. The stress levels are reported to be higher among the urban population due to longer working hours, schedule complexities, social isolation, and lack of support at the professional or personal front [7]. In India, the prevalence of DM was widely believed to be related to stress due to physical inactivity, dietary changes, and unhealthy lifestyle conditions [1]. In this respect, the studies related to stress are essential as it is often found associated with numerous chronic disorders [8].

Psychological stress is believed to be an essential risk factor for DM, and stressful experiences may affect the onset and metabolic control of DM. A large population-based survey of glucose tolerance found an association between stressful experiences and the diagnosis of type 2 DM [9]. Previously, it was found that stress-related factors, e.g., stressful workplace or traumatic life events, depression, type A personality, mental health problems, can independently be responsible for DM. In recent years, the potentially debilitating effects of stressful experiences on poor blood glucose control and the development of diabetic complications have been studied. High emotional distress and depression are also linked with DM [10]. This is a complex area with fewer studies being done in adults with type 2 DM [11]. The chronic stress factors can over-activate the hypothalamic-pituitary-adrenal (HPA) axis, resulting in an increased release of various insulin counter-regulatory hormones such as cortisol and adrenaline [10] and eventual derangement in the metabolic control of DM. For example, elevated plasma catecholamine levels and glucose intolerance have been found to be associated with stress even in healthy individuals [12], suggesting that stress can lead to transient hyperglycemia in the non-DM population as well.

In a previously published study, it was noted that psychological stress mobilizes glucose and lipid release into the circulation with increased production of cytokine. Moreover, chronic or repeated stressors can lead to dysregulated glucose metabolism, neuroendocrine function, and low-grade inflammation. Furthermore, psychological stress can adversely affect health behaviors such as food choice, medication adherence, physical activity, contributing to type 2 DM risk. Patients with established DM were also found to have poor glycemic control and cardiovascular complications due to depression and DM-related distress [13].

Many studies point towards the peculiar features in Indian population that are responsible for increasing the susceptibility to DM [14]. For example, the genetic susceptibility to DM is stronger in Indian subjects, and it is thus essential to examine the role of stress in exacerbating DM. Twins from a family of those with diabetes provide an interesting piece of evidence to show that stress indeed plays a vital role in the pathogenesis of type 2 DM [15]. Therefore, it is crucial to examine further the association of stress with DM using a larger population sample before any new public health intervention is planned. The nationwide data in this respect is still lacking. The present study was, therefore, planned to analyze the association between perceived stress category and blood glucose markers stratified by diabetes staging.

## Material and Methods

### Study design

The current descriptive study was a subset analysis of the previously published nationwide cross-sectional survey conducted on 16,368 participants, using a multilevel stratified cluster sampling technique with random selection, among urban and rural populations covering all Indian zones of the country. The study was conducted under the National Multicentric Diabetes Control Program, also called the Niyantrita Madhumeha Bharata Abhiyaan (NMB). This was funded by the Central Council of Research in Yoga and Naturopathy, New Delhi (CCRYN), and implemented by the Indian Yoga Association (IYA).

### Screening of participants

The Indian population was divided into seven major zones according to geographic distribution viz. North, South, East, West, North East, North West and Central. In each zone, participants from both the urban and rural regions were recruited. Participants were screened and selected from the general population based on the defined inclusion criteria: participants with hypertension and obesity; and exclusion criteria: participants with cardiovascular problems or who had undergone any major surgery.

Door to door screening was carried out through the Indian Diabetes Risk Score (IDRS) that is comprised of questions related to the two modifiable (physical activity and waist circumference) and two non-modifiable (age, family history) factors. Both male and female participants with an IDRS score of above 50, who fall under the category of high risk for DM, were further called for registration. In a previous study, Pawar et al. showed the sensitivity of IDRS as 73% and specificity 58.7% at a cutoff of >50 [16]. DM patients who were recruited took standard antidiabetic drugs for their glycemic control (Figure 1).

### Registration and Recruitment

From the selected population, only those participants who had a higher IDRS score were enrolled. Based on the self-declared DM status, they were subjected to a biochemical and psychological assessment. The DM or prediabetic status was determined using a combination of both self-report and biochemical results. The complete description of the research methodology, including study design, sampling strategy, study methods and quality assurance, data collection, assessments, data compilation, has been published previously in detail [17].

### Biochemical Assessment

The biochemical determinants of DM were estimated by an accredited diagnostic laboratory using standard diagnostic procedures. Blood samples were collected at selected centers in seven zones throughout the country. The glycated hemoglobin (HBA1c), fasting blood sugar (FBS), and postprandial blood sugar (PPBS) were estimated. The diagnostic protocol for these tests was aligned to and standardized across the centers as all tests were done by branches of a reputed diagnostic laboratory.

### Psychological Assessment (Stress Level Analysis): Perceived Stress Scale (PSS)

The Perceived Stress Scale (PSS) is a widely used instrument for measuring stress levels of the general population with psychological disorders. It is a self-administered questionnaire with ten statements where the participants were asked about feelings and thoughts about their own lives in the past month, and they had to choose from 5 options (0-4), ranging from ‘never’ to ‘very often’. Of the ten questions, six statements measured the stress level, and the other four statements measured counter stress, i.e., the four counter stress questions assessed the level of confidence the person has while facing a stressful situation, and these are scored in the reverse order. The maximum achievable score was 40, which was divided into three categories (0-15: mild stress; 16-30: moderate stress; 30-40: severe stress). PSS-10 is a revised version of the original scale comprising of 14 items (PSS-14) and was found to be psychometrically comparable and as reliable as the original scale [18]. Data curation was ensured, and only those individuals who had answered all the questions related to the PSS questionnaire were included in the study.

### Data Analysis

Data analysis was accomplished by applying the Pearson correlation test, paired sample t-test, chi-square test, and one-way ANOVA by using SPSS (21.0). P≤0.05 was taken as statistically significant.

### Ethical considerations

All subjects were informed about the aim of the research, and their written informed consent was obtained. Ethical permission was obtained from the Institutional Ethics Committee (IEC) meeting held at Morarji Desai National Institute of Yoga vide reference no. RES/IEC-IYA/001 dated 16th Dec 2016.

## Results

### Socio-demographic details

The current study analyzed a total of 1134 participants, from the previously published study with prediabetes (n=649) and DM (n=485) in order to evaluate the association of perceived stress with their glycemic changes [19].

### Association between blood glucose parameters and perceived stress in different populations

Based on the PSS score, people with DM and prediabetes were divided into a minimum, moderate, and severely stressed population. FBS levels were found to be marginally (p=0.08) higher in the severely stressed (109.80 ± 25.76) people with prediabetes, than moderately (99.24 ± 18.97) and minimally stressed (99.49 ± 17.79) populations. Additionally, PPBS levels were found to be marginally (p=0.07) higher in the severely stressed (155.5 ± 40.31) people with prediabetes than the moderately (121.57 ± 37.50) and minimally (121.23 ± 36.95) stressed population. Though alterations in FBS and PPBS levels were not significant among the three categories, HBA1c levels were found to be significantly (p=0.008) higher in the severely stressed (6.12 ± 0.27) people with prediabetes compared to moderately (5.97 ± 0.21) and minimally stressed (5.95 ± 0.22) population (Table 1).

**Table 1:** PSS score categorization into minimum, moderate and severe in people with prediabetes and its association with glycemic parameters.

Blood Parameter	Stress Category	N	Mean	Std Deviation	p-value
FBS (mg/dl)	Minimum	307	99.49	17.79	0.08
	Moderate	277	99.24	18.97	
	Severe	10	109.80	25.76	
PPBS (mg/dl)	Minimum	174	121.23	36.95	0.07
	Moderate	190	121.57	37.50	
	Severe	4	155.5	40.31	
HbA1c (%)	Minimum	328	5.95	0.22	0.008
	Moderate	308	5.94	0.21	
	Severe	13	6.12	0.27	

Similarly, glycemic changes were also noted in the DM population. The mean FBS levels were found to be significantly (p=0.02) higher in the severely stressed population (239.28 ± 99.52) than the minimally (171.69 ± 76.78) and moderately (182.15 ± 72.35) stressed population. However, PPBS (p=0.73) and HBA1c (p=0.19) levels did not show any significant difference between the different categories (Table 2).

**Table 2:** PSS score categorization into minimum, moderate and severe in the diabetic population and its association with glycemic parameters.

Blood Parameter	Stress Category	N	Mean mg/dl	Std Deviation	p-value
FBS (mg/dl)	Minimum	247	171.69	76.78	0.02
	Moderate	166	182.15	72.35	
	Severe	7	239.28	99.52	
PPBS (mg/dl)	Minimum	164	236.24	101.35	0.73
	Moderate	140	264.20	107.42	
	Severe	3	256.66	44.65	
HbA1c (%)	Minimum	273	8.55	1.90	0.19
	Moderate	204	8.74	1.98	
	Severe	8	9.46	3.03	

## Discussion

The current study provides a comprehensive comparison of the effects of perceived stress on the blood glucose parameters among people with prediabetes and DM in the entire Indian population. Stress has been studied in relation to various diseases and their course of progression and is found to be a risk factor in many disorders. The present study has shown that people with prediabetes are under more stress as compared to those with diabetes in India. Stress is believed to be closely related to DM because both have common risk factors such as inadequate eating behaviors, sedentary lifestyle, smoking, and alcohol abuse [20]. Also, chronic stress reactions and depression affect the hypothalamic-pituitary-adrenal (HPA) axis leading to abdominal obesity, another risk factor of DM [21, 22]. Another explanation for the correlation of stress with DM is evidenced by immune system alterations as a result of stress. Pro-inflammatory cytokines and glucocorticoids such as cortisol have been found to be elevated in response to chronic stress [23]. Stress and its correlation to the onset of DM have been described earlier in which the retrospective analysis of 25 adult DM patients was reported to have a history of antecedent stress [24]. In a follow-up study, it was found that stressed men, but not women, were two times more likely to develop DM. Another intriguing finding of the study described that participants who reported high levels of stress were less likely to quit smoking or drinking and displayed an increased tendency for physical inactivity. Both these factors are known risk factors for type-2 DM [20]. Interventions such as healthy lifestyle modifications have earlier been proved to be effective in regulating DM progression. One hundred fifty minutes of physical workout per week reduced the incidence of DM by 58% [25]. The Finnish Diabetes Prevention Study was the first intervention study to prevent or postpone the occurrence of DM in high-risk individuals. The follow-up studies reported that the intervention group underwent dietary and physical activity-related lifestyle modifications and showed significantly greater improvement in weight reductions and glycemic index [26].

Another study in which medical conditions (DM and lifestyle factors) were self-reported by individuals in the form of a questionnaire at the baseline, 5, and 10 years later, explored and proved the association of perceived mental stress with the onset of DM [27]. Similarly, Toshihiro and co-workers, after a 3-year follow-up, showed that impaired glucose metabolism (risk factor of DM) was related to stress [28].

A healthy individual under stress initially begins with a primary flight or fight response against the stressor, but when the stress is prolonged, the body sets up a resistance phase resulting in hormonal, metabolic, and physiological changes. In the people with prediabetes, the body appears to be in the adaptation phase, physiologically.

Psychological stress is involved in the progression of multiple diseases. Severe types of psychological stress affect both the nervous and peripheral systems. Much of experimental evidence suggests that the severity of the disease depends upon the course and duration of stress. It has also been observed that when the person encounters stress for the first time, it targets the nervous system with alteration in pathophysiology and immune system [29]. Physiologically, stress activates the endocrine system, which produces the primary effector (cortisol) regulating the vast range of physiological systems, including the immune and cardiovascular systems, gluconeogenesis and protein, carbohydrate, and fat metabolism [30].

However, it is still debatable whether stress is responsible for the progression of systemic diseases or disease progression leads to psychological stress or both [11]. There are studies that correlate stress with DM, i.e., those with DM are more distressed because of the perception of complications they anticipate. DM treatment or diagnosis can be one of the stressors as these patients have to keep up with the sugar levels by following a strict diet regime [10]. Therefore, it is not very clearly understood whether stress is a cause or a consequence of DM [31]. Our study does not provide any evidence in this regard, and in order to test this discrepancy, large randomized controlled trials are required with a longitudinal follow-up.

## Conclusion

People with prediabetes and severe perceived stress have an increased HbA1c level, which calls for a comprehensive analysis with a longitudinal study of the role of perceived stress in the progression of prediabetes into DM. Additionally, higher FBS levels in patients with DM and severe perceived stress suggests the need for establishing comprehensive diabetic care inclusive of stress management.

## Acknowledgments

The authors would like to acknowledge the volunteers of Niyantrita Madhumeh Bharat for their help in data collection, Dr. Rama Malhotra and Dr. Suchitra for data analysis. Abdul Ghani for data digitization, CCRYN for providing support regarding human resources, MOHFW for providing financial help for the cost of investigations, IYA for the overall project implementation, and Dr. Deepti, Head of the English Department, Panjab University, for proofreading.

## Source of Funding

This research work was supported by the Ministry of AYUSH, Government of India.

## Conflict of interest

The authors declare that there is no conflict of interest.
